# Non-Invasive Biomarkers of Musculoskeletal Health with High Discriminant Ability for Age and Gender

**DOI:** 10.3390/jcm10071352

**Published:** 2021-03-25

**Authors:** Sandra Agyapong-Badu, Martin B. Warner, Dinesh Samuel, Vasiliki Koutra, Maria Stokes

**Affiliations:** 1 School of Sport, Exercise and Rehabilitation Sciences, University of Birmingham, Birmingham B15 2TT, UK; 2 School of Health Sciences, University of Southampton, Southampton SO17 1BJ, UK; m.warner@soton.ac.uk (M.B.W.); D.Samuel@soton.ac.uk (D.S.); V.Koutra@soton.ac.uk (V.K.); M.Stokes@soton.ac.uk (M.S.); 3 Centre for Sport, Exercise and Osteoarthritis Research versus Arthritis, Southampton SO17 1BJ, UK; 4 Southampton Biomedical Research Centre, Southampton SO16 6YD, UK

**Keywords:** ageing, musculoskeletal health, physical function, physical frailty, screening

## Abstract

A novel approach to ageing studies assessed the discriminatory ability of a combination of routine physical function tests and novel measures, notably muscle mechanical properties and thigh composition (ultrasound imaging) to classify healthy individuals according to age and gender. The cross-sectional study included 138 community-dwelling, self-reported healthy males and females (65 young, mean age ± SD = 25.7 ± 4.8 years; 73 older, 74.9 ± 5.9 years). Handgrip strength; quadriceps strength; respiratory peak flow; timed up and go; stair climbing time; anterior thigh tissue thickness; muscle stiffness, tone, elasticity (Myoton technology), and self-reported health related quality of life (SF36) were assessed. Stepwise feature selection using cross-validation with linear discriminant analysis was used to classify cases based on criterion variable derived from known effects of age on physical function. A model was trained and features selected using 126 cases with 0.92 accuracy (95% CI = 0.86–0.96; Kappa = 0.89). The final model included five features (peak flow, timed up and go, biceps brachii elasticity, anterior thigh muscle thickness, and percentage thigh muscle) with high sensitivity (0.82–0.96) and specificity (0.94–0.99). The most sensitive novel biomarkers require no volition, highlighting potentially useful tests for screening and monitoring effects of interventions on musculoskeletal health for vulnerable older people with pain or cognitive impairment.

## 1. Introduction

The use of large-scale pooled analyses and data sharing is a potential source to generate evidence to address complex scientific challenges and develop strategies to achieve healthy ageing. However, the success of such analyses depends on robust measures of health in ageing. Longitudinal ageing studies like The English Longitudinal Study of Ageing (ELSA), The Irish Longitudinal Study of Ageing (TILDA), and the Cardiovascular Health study in the USA have utilised different measures of physical function to provide evidence of early predictors of later declines in health, which may be useful in public health and clinical practice [1]. The World Health Organisation defines healthy ageing as ‘the process of developing and maintaining the functional ability that enables wellbeing in older age’ [2]. A Lancet report looking at trends in ageing studies highlighted the need for studies that focus on refining measurements of health, functioning and disability in older people, how these measures evolve and their subsequent effect on the health-care system and their associated economic implications [3]. Such studies could be enhanced by the use of robust measures that are predictive of physical performance in healthy ageing. Measures of physical function serve as an indicator of health status and in later life, lower limb physical function is predictive of morbidity, loss of independence, mortality, healthcare cost and utilisation [4,5,6]. However, the success of physical performance assessment is reliant on the ability of the individual to comprehend the tasks involved. In the presence of cognitive impairment or pain from underlying conditions (e.g., arthritis), it may be challenging to assess physical function, and the ability to generate an indicator of function in this group of older adults is of great benefit to geriatric assessment and ageing research.

Hirsch et al. [7], highlighted that the rate of performance change may be more useful than a single, cross-sectional measurement for estimating disability risk. Using a combination of tests with a holistic approach to physical performance assessment may provide better discrimination between specific groups of individuals, particularly to guide performance change from an at-risk category into frailer categories. The present study focused on using a novel approach to ageing studies by means of discriminant analysis to identify which measures of performance have high discriminant ability to classify healthy older adults based on their age and gender, which can be indicative of musculoskeletal (MSK) health. Older adults have poorer physical performance and MSK health, thus using these known effects of ageing on MSK health, the study sought to identify which combination of tests has high discriminant ability to show these changes in MSK health with ageing. This is because age/gender really can be related to or indicative of MSK health. Its intention was to contribute towards potential means of predicting future health and survival, as well as enabling examination of older adults across the spectrum of different functional abilities. The battery of tests included conventional and novel tests reporting upper and lower limb function, self-reported quality of life, respiratory function, muscle size and mechanical properties in healthy young and older community-dwelling adults. The battery of tests selected included simple, reliable and portable technologies, due to a lack of such tools suitable for field-testing to assess physical health status. 

### 1.1. Aim

To use the known effects of age on (MSK) health to test the discriminant ability of a combination of routine physical performance tests and novel measures to classify healthy individuals according to their age and gender. 

### 1.2. Objectives

1. To use a novel application of linear discriminant analysis including stepwise feature selection using leave-one-out cross validation in rehabilitation research to classify healthy adults accurately into four classes according to age and gender.
2. Provide a battery of simple, robust, non- invasive dry biomarkers indicative of MSK health for use in ageing studies.

## 2. Experimental Section

### 2.1. Participants 

This cross-sectional observational study recruited healthy individuals from the community. Participants included 138 community-dwelling healthy young and older people in Southampton, UK (young; 37 men, 28 women; and older 30 men, 43 women). Mean age was young 25.7 ± 4.8 years and older 74.9 ± 5.9 years. 

Young participants (18–35 years) were recruited from the University. Participants who took part in sports, exercised above moderate levels (more than three times per week), or competitively at university level or above were excluded. 

Older adults (65 ≥ 90 years) were sedentary to moderately active based on the Physical Activity Scale for the Elderly (PASE) score [8,9], cognitively aware and able to understand the study procedures. Screening ensured the older group was representative of the older population by including individuals with common medical conditions, such as hypertension and diabetes, so long as these conditions were well controlled and the participants medically stable. 

All participants were encouraged to refrain from drinking alcohol for 24 h before each visit, and from taking part in strenuous physical activity for 48 h prior to assessments. Cognitive impairment was not assessed objectively but the lead investigator (S.A.-B.) made a clinical judgement of the participant’s ability to understand the study requirements and instructions during the recruitment process. Participants were asked if they received any form of care, any mobility challenges and a discussion on their ability to attend assessment sessions independently. Participants provided written, informed consent prior to testing and ethical approval was obtained from the local Ethics Committee (FoHS-ETHICS-2011-060). 

### 2.2. Physical Performance Assessment

#### 2.2.1. Handgrip Strength

Grip strength was measured using a JAMAR hydraulic dynamometer, with the handle set in the second position. Participants were tested in a seated position, with shoulder adducted and in neutral rotation, elbow flexed to 90° and forearm positioned neutrally resting on a support [10].

#### 2.2.2. Quadriceps Strength

A purpose-built rig consisting of a chair, force transducer (Advanced Force Torque Indicator, Mecmesin Ltd., Horsham, UK) and strain gauge was used to measure the isometric strength at the knee in sitting. A surf strap was attached just above the lateral malleolus and connected to the strain gauge bar. The output from the transducer was amplified using a strain gauge amplifier and was input into a 16-channel analogue to digital data collection system, housed inside a computer equipped with turbo Pascal program with a sampling frequency of 50 Hz per channel [11]. Participants were instructed to maintain their knees and hips at 90 degrees and this position was secured using a strap across the pelvis to minimise the extraneous body movements and isolate force measurements to the quadriceps muscles as much as possible. Contractions were isometric, so there was no movement as the participant attempted to extend their knee against the inextensible strap above the ankle. A sub-maximal practise was performed prior to actual testing of three maximal isometric quadriceps contractions for a duration of 3 s each. A 30-s rest period was given between consecutive contractions and the maximum value out of three trials was used in the analysis [10]. 

During strength testing, visual feedback from the Jamar Hand Dynamometer and the Strain Gauge system was utilised to help ensure maximal efforts as much as possible. The investigator provided the same set of verbal instructions for all participants for consistency and provided audible encouragement. Variability between efforts provided an indication as to whether efforts were maximal, so this guided the investigator as to when more encouragement was needed. 

#### 2.2.3. Peak Flow

Peak flow was performed in standing using a handheld Clement Clarke Mini-Wright Standard Range Peak Flow Meter; EN13826/EU (Clement Clarke International; Essex, England). A disposable cardboard mouthpiece was inserted in the meter and the pointer set at zero (L/min) position at the start of each trial. Participants held the meter horizontally, positioning fingers to stay clear of the scale and slot. They were instructed to take a deep breath, place the meter in their mouth, closing their lips around the mouthpiece and then blow as hard and as fast as they could. Participants were encouraged to perform at their maximal effort. The maximum reading out of three is reported [12]. 

#### 2.2.4. Timed Up and GO

This test involved a standardised protocol of rising from a chair, walking 3 m at usual pace, turning and walking back along the walkway and sitting down. Participants completed a practise trial before the timed trial began. Timing started when participants stood up and stopped when they sat back down. The fastest time measured in seconds out of three trials was used for analysis [13].

#### 2.2.5. Stair Climbing Capacity

The participants were asked to climb a stairway with 11 steps at their own pace, with or without the handrail and only to stop if they experienced exhaustion, limiting dyspnoea, leg fatigue, or chest pain. Timing began when participants lifted their foot for the first step and ended when both feet returned to the landing at the base of the stairway [14]. 

#### 2.2.6. Anterior Thigh Thickness (Ultrasound Imaging; USI)

Anterior thigh thickness was assessed using a real-time ultrasound scanner (Aquila; Esaote Spa, Genova, Italy) with a 6-MHZ linear transducer array (60-mm footprint) to take B-mode cross-sectional images of the quadriceps muscle (rectus femoris and vastus intermedius) over the anterior mid-thigh. The lead investigator S.A.-B., who had already established intra-rater reliability, took two images at two-thirds of the distance between the anterior–superior iliac spine and the superior pole of the patella in the sagittal plane of the dominant limb [15,16]. Muscle thickness and non-contractile tissue thickness (subcutaneous adipose tissue and peri-muscular fascia) were measured offline using a MATLAB algorithm written by one of the authors (MW).

#### 2.2.7. Muscle Mechanical Properties 

The MyotonPRO device was used to assess biceps brachii (BB) and rectus femoris (RF) muscle stiffness, tone and decrement (elasticity). A standardised protocol for using this hand-held device was followed [17]. The device elicits oscillations of muscle after a probe applies a brief mechanical impulse following a constant pre-load to the skin over the muscle. From these oscillations, the device quantifies various parameters simultaneously, including non-neural tone and mechanical properties such as dynamic stiffness and decrement [18]. The frequency determined by fast Fourier transform (FFT) which was most characteristic in the registered oscillation acceleration signal, indicates resting tone or state of tension of an activated muscle (Fmax; (Hz)). Stiffness (N/m) is a measure of the muscle’s ability to resist an external force that modifies its shape, the higher the N/m value, the stiffer the muscle [17]. Logarithmic decrement describes the tissue’s ability to restore its shape after being deformed, and defined as the dissipation of mechanical energy in the tissue during an oscillation cycle. The smaller the decrement value, the smaller the subsequent dissipation of mechanical energy and the higher the elasticity [17]. 

#### 2.2.8. Self-Reported QoL (SF-36)

Participants completed SF-36 to assess the quality of life. The questionnaire has good reliability for use in community dwelling older adults [19].

### 2.3. Assessing Reliability for Battery of Tests

The lead investigator performed all tests and demonstrated good to excellent test–retest reliability in assessing participants using this battery of tests. Intraclass correlation coefficients (ICC 3,1) for tests repeated on different days were: hand grip strength (ICC 0.86–0.97), quadriceps strength (0.87–0.92), peak flow (0.90–0.91), timed up and go (0.54–0.71), stair climbing (0.51–0.93), muscle mechanical properties (biceps brachii: 0.72–0.93; rectus femoris: 0.68–0.94), anterior thigh thickness measurements using ultrasound (0.88–0.97). 

### 2.4. Statistical Analyses 

The data were grouped using age and gender (indicators of physical function) into four classes (i.e., young males, young females, older males, and older females). This categorical variable was used as the criterion (dependent) variable for the classification. Stepwise feature selection using cross validation with linear discriminant analysis was implemented in the R (R Core Team, 2021; Vienna, Austria) statistical programming language [20]. Leave-one-out cross validation was used both in feature selection and to assess the classification skill of the final model as it is approximately unbiased and reduces overfitting compared to the use of hypothesis testing-based feature selection.

In particular, for every model considered, formed from a subset of features, for each training/test split in cross-validation, the model was trained on the training fold and the predictive performance (correctness rate) was calculated on the test fold. The correctness rate was then averaged across all test folds, to get an overall predictive measure for that model. We chose the model with the best cross-validated performance. Once the model was developed, a leave-one-out cross validation approach was used to assess the ability of the model in classifying the participants.

## 3. Results

### 3.1. Participant Characteristics and Absolute Values for Battery of Tests

The majority of young adults were in the lower age band (18–22 years; *n* = 23), and majority of older adults were in their mid-seventies (75–79 years; *n* = 24). Participants had moderate to high self-perceived QoL (SF36), physical function declined with age in both males and females (Table 1). 

### 3.2. Classification Using Features from Battery of Test 

Features from novel tests were combined with conventional tests for the classifier and the model was trained and features selected using 126 complete cases out of 138 cases. Twelve cases (9%) were excluded due to at least one missing discriminating variable. The model yielded an accuracy of 92% (95% CI; 86–96%). The final classification model included the features peak flow, timed up and go, BB decrement, anterior thigh muscle thickness and percentage thigh muscle. Table 2 presents the cross-tabulation of observed and predicted classes (obtained via leave-one-out cross validation). 

### 3.3. Classification Performance Parameters 

Table 3 shows some performance measures for the final model. High sensitivity (82–96%) and specificity (94–99%) were obtained for all four classes. The rates of true positives (positive predictive value; PPV) and true negatives (negative predictive value; NPV) were also high, except for class 2 where a number of false positives occur. 

### 3.4. Misclassified Cases

**Figure 1:**
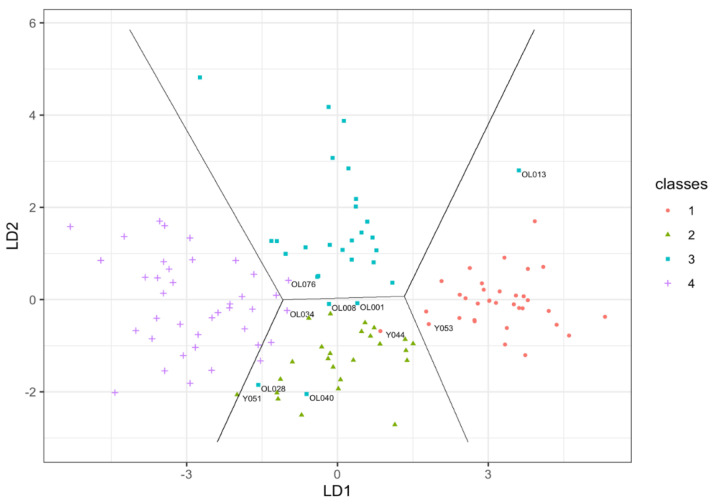
Decision boundaries from the linear discriminant analysis in the two-dimensional subspace spanned by the first two discriminant functions (LD1 and LD2) with misclassified cases labelled. Predicted classes indicated by colour: red; young males, green; young females, blue; older males, lilac; older females.

The model predicted ten misclassified cases comprising of 3 young and 7 older adults. Details of the misclassified cases are presented in Table 4 to show how these vary from class means. A plot of the first two discriminant functions shows a clear discrimination of all four classes into respective zones (Figure 1). Misclassified individuals are identified.

### 3.5. Effect of Body Mass Index on Classification Model

**Figure 2:**
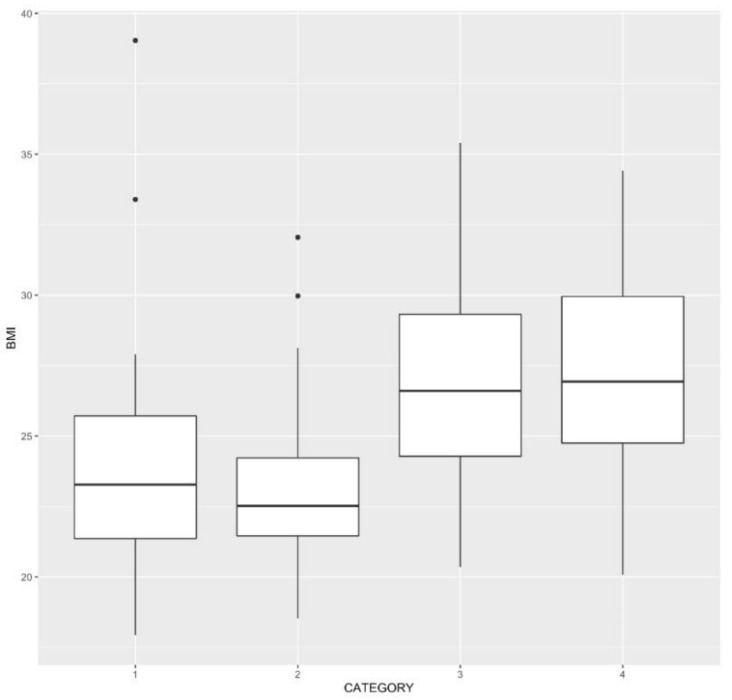
Boxplots of BMI and four categories; 1 = Young male, 2 = Young female, 3 = Older male, 4 = Older female. The whiskers of the boxplots extend to 1.5 times interquartile range for each category. Data beyond the end of the whiskers are plotted individually.

Boxplots of BMI for each of the four categories (Figure 2), indicate BMI being predictive of age but not gender. In addition, simple multinomial logistic modelling (not presented) and correlations between BMI and the other variables (Table 5) supported this finding. 

## 4. Discussion

The stepwise feature selection has identified important features with high discriminant ability using participants from known age and gender categories, which serve as proxy indicators of MSK health. The important contribution of the present study is the discriminant ability (for age and gender) of the comprehensive battery of objective tests, providing evidence for using these tests to assess MSK health in healthy ageing. The choice of portable technologies suitable for field-testing, including conventional and novel technologies to assess physical health status, as well as producing reference data for healthy young and older adults, may prove to be an important addition to ageing research. Overall, these results show that combining novel and conventional tests enabled the training and testing of a model to classify majority of cases into their respective classes with a low error rate. The relatively novel technologies for assessing physical performance (MyotonPRO and USI assessment of thigh composition) have therefore contributed to the successful classification model of individuals, which otherwise would not have been as robust. Consequently, a key addition to the findings of this study is the inclusion of novel tests that are less burdensome or rely on little or no volition e.g., Myoton to assess the physical function in healthy ageing. 

It is well recognised that the sensitivity of tests of MSK health varies to changes with ageing and no single test represents the whole body, so there is a need for a combination of tests for assessing MSK function with ageing. Another important consideration is finding tests that can be used in community settings, away from laboratories and hospitals. Although hand grip strength is considered the most representative marker of MSK health, it is known that it does not, for example, reflect lower limb strength [10,21], which is vital to maintaining physical independence. The present study addressed the need to identify suitable tests by examining combinations of simple, rapid, portable tests, intentionally studying groups for age and gender with known differences in MSK function, as a first step. The present results confirm the known effects of ageing on physical performance to highlight which tests may be most indicative of age-related changes and appropriate for assessing MSK health status. Many studies of health and ageing rely on physical functioning and disability measures to indicate health [22], and in keeping with this finding, most of the physical function tests in the present study showed significant gender and age-related differences in MSK health. The novelty of this study was to then build on these differences and further reduce the variables used to assess MSK health status. The classification approach allowed further reduction of the number of features by identifying which combination of tests had the highest discriminant ability to classify the participants into categories of MSK health using age and gender. The power of this model is confirmed by the high sensitivity (82–96%) and specificity (94–99%), demonstrating the potential of using these tests for assessing MSK health. 

The ability of the model to categorise participants using the selected features is in keeping with the age and gender physiological and morphological changes in MSK features [23]. The five features selected (anterior thigh muscle thickness, percentage thigh muscle, timed up and go, biceps brachii elasticity and peak flow) may be interrelated in their contribution to functional independence. For instance, greater thigh muscle strength and thigh muscle mass were associated with decreased risk of mobility disability and a slower decline in gait speed and function in older people [24,25]. A higher body mass index (BMI) and percentage body fat were associated with poor physical function [26]. Similarly, conservation of muscle mass and strength in thigh muscle has reported consequences for survival in old age [27].

Peak flow and walking speed, on the other hand, have been highlighted as indicators of robustness and are independent predictors of health-related outcomes [28]. Likewise, age-related change in muscle elasticity was associated with walking ability and physical function in community-dwelling individuals [29]. Furthermore, age-related decrease in rectus femoris [30], and gender differences in biceps brachii elasticity [31] have also been demonstrated. The reported contributions of these outcome measures to indicating functional independence may explain their inclusion in the final model representing MSK health for the present study. Consequently, these findings indicate that further investigation is warranted to include more age groups and people with different levels of physical activity to assess the robustness of the final model.

With regards to the classification approach used, the model was trained and features selected using 126 samples. Once the model was developed a leave-one-out cross validation approach tested the skill of the model for out of sample classification, achieving 92% accuracy. Cross validation was employed as part of the stepwise feature selection to prevent overfitting. Leave-one-out cross validation was chosen as it directly reflects the final assessment of the model and the clinical interest in classification of individual cases (see Table 2 and Table 3). The final model includes features from a range of measures that may be useful to consider when assessing the effects of ageing on MSK health. The stepwise procedure identified respiratory peak flow, walking speed, upper limb muscle elasticity and anterior thigh muscle integrity (thickness and percentage muscle) as being important to discriminate with high sensitivity (82–96%) and specificity (94–99%) for all four classes. Consequently, these assessments may be considered a useful addition in an optimal toolkit for assessing MSK health as a marker of functional independence in healthy ageing. 

The high level of precision found may be due to the large phenotype difference between groups (Table 1) and several factors may alter the level of precision, which need to be investigated by building on the present database to include more age groups and people with different levels of physical activity, as well as patient groups, to enable assessment of individuals. The approach used in the present study could be further explored to identify which aspects of physical performance are functioning poorly, to aid targeted interventions. Practically, once the age and gender of an individual are known, an assessment using this battery of tests could provide an indication of their MSK health to determine whether this is in agreement with their biological age. This approach will provide valuable objective information on biomarkers for physical function, which in turn could be a useful indicator of functional independence to aid in categorising at-risk individuals to facilitate timely preventative action. 

When details of the misclassified cases from the four classes were scrutinised, the data showed that older adults who were misclassified into a higher performance group such as young adults had better respiratory peak flow, thicker anterior thigh muscles, more elastic upper arms and faster walking speed compared to age-matched counterparts. Only one young female was misclassified as an older female, and a lower respiratory peak flow and lower anterior thigh muscle thickness accounted for this. The misclassification is an interesting outcome from a practical perspective in terms of identifying individuals who had better function than expected for their age and/sex. Identifying which features were able to detect better or poor MSK health would be useful in monitoring effects of treatment as MSK health improves, highlighting the potential of their use in clinical practice. The findings in this study sample from the general population highlight the interplay of these biomarkers to achieve optimal physical function for functional independence. Therefore, in healthy ageing, not only is muscle strength important but the muscle quality (mechanical property) as well as amount of non-contractile tissue are equally useful for optimal MSK function. The misclassified cases who appear healthier than predicted support the need for studies of older people with different activity levels such as older golf players [32], to provide reference values for appropriate assessment. For instance, when an older active person is injured, their rehabilitation goals need to be relevant to their activity level as opposed to age-matched older sedentary person and these tests can provide objective information on effect of the rehabilitation process.

The potential of adopting this classification approach to objectively classify community-dwelling individuals based on their physical function is relatively novel compared to other approaches to discriminate movement faults of the scapula [33], or cyclical body bends among Parkinson’s disease patients [34]. The level of accuracy recorded for classification model in the present study is high, compared to some of the published levels for discriminant analysis to categorise older adults based on different criterion features. Recently a study reported accuracy of 73% for five predictor variables including cognitive function to identify individuals at high risk of lapses once engaged in regular physical activity [35]. A similar lower accuracy of 78.2% was reported using five predictor variables for detecting individuals at high risk of depression among community-dwelling older adults [36]. In another study, a slightly higher accuracy of 82.4% was reported using features extracted from breathing patterns along with common clinical variables to detect Periodic Breathing in older adults [37]. It is possible that a combination of linear discriminant analysis with principal component analysis may provide higher accuracies, but the present study selected features that were reliable and showed statistically significant differences between classes. 

Chatterji et al. [3], reported that in addition to focusing on identifying trends in the prevalence of chronic diseases in older adults, functioning-based assessments of health status need to become an integral part of national data collection efforts to monitor trends in healthy life expectancies, especially for older adults. A recent review has highlighted the absence of such core measures for use in interventions that improve physical outcomes in pre-frail and frail older adults [38]. With further testing, the present battery of tests could contribute to physical health assessment by way of this comprehensive MSK health measurement tool kit for future ageing studies to aid data sharing across continents towards improving or understanding the mechanisms of ageing and developing strategies for managing physical decline.

The five most relevant features in the model are from four assessments (peak flow, walking speed, muscle mechanical properties, and anterior thigh muscle thickness) which may seem appropriate for research trials and could be explored clinically for diagnosis and monitoring. Evidently, two of these require little or no volition, are feasible to use in older adults, reliable, valid, simple, easy and quick measures that can be performed routinely. Additionally, training required to use the technologies is not laborious and relatively cheaper compared to MRI and emerging technologies like shear wave elastography. We recommend a battery of tests with these categories of assessments; respiratory function, walking speed, anterior thigh muscle thickness (USI imaging) and muscle mechanical properties (MyotonPRO assessment) for assessing MSK health. These tests may be further investigated with longitudinal studies to assess its suitability for screening purposes in community settings to classify older people in the pre-clinical frailty stage to aid in the timely prevention and management of frailty. To achieve this, further studies of people with frailty are needed to determine the potential use of this classification approach.

The present battery of tests could also be an excellent addition to physical frailty assessment particularly in intervention studies for vulnerable older people with pain or cognitive impairment to identify which additional aspects of MSK health influence frailty in this age group. Screening tests for detection of signs of premature frailty would potentially have major health and social care cost savings, if preventive measures are established and efforts to restore function with rehabilitation employed. Knowing which physical frailty indicators predict mobility-disability is useful to identify older adults who might benefit from an intervention that prevents disability or increases functioning in daily life. Therefore, a practical implication of this research includes the use of these biomarkers to pragmatically screen physical health status of older people in clinical or community settings, to classify those at the pre-clinical frailty stage and facilitate prevention and management. A future focus of research could be to assess the prognostic ability of the battery of tests for disability and mortality in older people. 

### Limitations

The generalizability of the present study to the older community-dwelling population may be limited due to the high proportion (79%) of the group being under 80 years i.e., younger old rather than older old. The present sample size of 138 is relatively small but provides a basis for larger studies for more robust modelling. 

Leave-one-out cross validation does alleviate (although does not prevent) over-fitting, particularly compared to stepwise hypothesis testing and for small or moderate sized data sets, it is also attractive, as then even small changes to the training data may result in large and unrepresentative changes to the fitted model. Further splitting into training, testing and validation sets may help and future studies with larger numbers should consider this approach.

The cross-sectional nature of the study makes assessment of the impact from other prevailing health conditions that could affect performance challenging. However, data from classification analysis showed that the misclassified older adults reported high PASE scores and three of them were subsequently classified as young adults. This presupposes that older persons who were currently physically active, or had been in the past, were likely to record better physical performance. 

While fat thickness may be potentially associated with muscle parameters in certain cases [39], it is difficult to conclude that fat thickness influences muscle parameters directly due to inconsistences across age and gender groups [40]. A recent study in healthy participants reported that probe positioning away from the muscle midpoint, alteration in muscle length, level of contraction, and prior physical activity significantly altered mechanical tone and stiffness of biceps brachii and rectus femoris muscles [18]. In recognizing factors that influence muscle parameters, it is important to consider the technique utilised for assessing these to provide a context [41,42]. Future studies should assess the influence of factors such as differences in elasticity, tissue composition, hydration and the use of Myoton. 

A higher body mass index (BMI) and percentage body fat were associated with poor physical function [26], and more recently a promising new approach to improve the accuracy of age estimations would be to factor in body mass measurements, and also considering osteological markers of obesity [43]. A study in 40 sedentary men also demonstrated that lower limb tendon length did not increase significantly with an increase in the BMI, body fat mass, dominant leg body fat content, and fat-free mass index. The authors however observed a greater intensification for the thickness of the tendon with a significant increase in tendon stiffness [44]. These findings indicate BMI may potentially influence physical function outcomes and the strengths of associations observed. When BMI was included as a potential predictor in the LDA, it was not selected by the cross-validated stepwise procedure highlighting that in the present group of participants, BMI did not influence classification predictions. From further exploration, BMI appeared to be predictive of age but not gender. Future studies in a larger sample are warranted to elucidate these findings.

Although investigation of comorbidities and medication use may provide further insight, unfortunately summary level data are only available, see Appendix A.

The present study did not report responsiveness of the battery of tests as a whole. However, responsiveness to change of the individual conventional and novel tests has been reported in the literature. For instance, in the use of Myoton testing of mechanical properties in upper-extremity rehabilitation for stroke patients, muscle stiffness was more responsive to change than tone and elasticity [45]. Similarly, responsiveness to change of muscle mechanical properties was reported in people with Parkinson’s disease following medication [46], and brain stimulation [47], physical treatment for back pain [48], and following physical activity [49]. The changes assessed using ultrasound imaging following functional electrical stimulation on muscle structure after stroke have also been reported [50]. Further research is now needed to investigate the responsiveness to change following an intervention of all assessments concurrently.

## 5. Conclusions

The classification approach demonstrated by the present study has advanced interpretation of data in rehabilitation research beyond the commonly used correlation and regression analysis techniques. The data provide evidence for future studies to assess the predictive ability of this battery of tests for physical function in a healthy cohort of older adults, preferably middle age to old age, to identify older adults at risk of frailty. These assessments could form a toolkit of standardised measurements for assessing MSK health in older adults. 

The most sensitive novel biomarkers require no volition, highlighting potentially useful tests for screening and monitoring effects of physical activity interventions or treatment as MSK health improves for vulnerable older people with pain or cognitive impairment. Older misclassified cases who appear healthier than predicted support the need for studies of older people with different activity levels, to provide reference values for appropriate assessment so rehabilitation goals are relevant to the individuals’ activity levels.

## Figures and Tables

**Table 1:** Participant characteristics and physical performance (*n* = 138; mean and standard deviation).

KERRYPNX	Young Adults Male (*n* = 36) Female (*n* = 27)		Older Adults Male (*n* = 32) Female (*n* = 43)	
Age (years)	24.9 ± 4.8	26.8 ± 4.6	74.1 ± 5.7	75.5 ± 5.9
Body mass index (kg/m^2^)	24.1 ± 4.1	23.2 ± 3.2	26.5 ± 3.5	27.1 ± 3.8 *
SF-36				
Physical function	97.4 ± 4.1	96.7 ± 6.8	90.7 ± 11	83.8 ± 16.8 *
Physical activity scale for the				
Elderly (PASE)	N/A	N/A	173 ± 69.7	142 ± 52.3 ^†^
Grip strength (kg)	45.3 ± 8.3	27.4 ± 5.2	35.7 ± 5.4	22.1 ± 4.6 *^,†^
Quadriceps strength (*N*)	514 ± 138	334 ± 101	343 ± 66	214 ± 54 *^,†^
Peak flow (L/min)	564 ± 80	395 ± 70	446 ± 109	307 ± 63 *^,†^
Timed up and go (s)	5.5 ± 0.9	5.3 ± 0.8	7.3 ± 2.4	7.7 ± 1.8 *
Stair climbing time (s)	7.1 ± 1.7	8.7 ± 2.8	11.8 ± 4.9	14.6 ± 5.1 *^,†^
Ultrasound Imaging				
Non-contractile tissue thickness (mm)	8 ± 3.4	14 ± 4.2	9 ± 4.5	16 ± 4.7 *^,†^
Muscle thickness (mm)	39 ± 7.5	29 ± 6.1	25 ± 4.4	20 ± 5.2 *^,†^
% Non-contractile tissue	17 ± 6	32 ± 7	26 ± 8	44 ± 7
% Muscle	83 ± 6	68 ± 7	74 ± 8	56 ± 8
Muscle mechanical properties			285 ± 58 15.7 ± 1.8 1.5 ± 0.4	291 ± 52 * 15 ± 2 * 1.6 ± 0.3 * 300 ± 44.4 *^,†^ 14.6 ± 1.9 ^†^ 1.6 ± 0.2 *
**Biceps Brachii**				
Stiffness (N/m)	214 ± 25	216 ± 28		
Tone (Hz)	14 ± 0.8	13.7 ± 1.2		
Decrement (log)	1 ± 0.2	1.1 ± 0.2		
**Rectus Femoris**				
Stiffness (N/m)	290 ± 39.5	231 ± 5.3	324 ± 31.4	
Tone (Hz)	16.2 ± 1.7	13.5 ± 1.3	16.5 ± 1.8	
Decrement (log)	1.3 ± 0.2	1.2 ± 0.2	1.6 ± 0.3	

Note: N/A = not applicable; * significant difference with age (2 tailed); ^†^ significant gender difference within age group; (*p* < 0.05).

**Table 2:** Cross-tabulation of observed and predicted classes.

Prediction	Reference			
	1	2	3	4
1	**32**	0	1	0
2	2	**24**	3	1
3	0	0	**23**	1
4	0	1	1	**37**

Classes; 1 = Young male, 2 = Young female, 3 = Older male, 4 = Older female. Bold numbers represent correctly classified cases in each class.

**Table 3:** Classification performance parameters (%).

	Class 1	Class 2	Class 3	Class 4
Sensitivity	94	96	82	95
Specificity	99	94	99	98
Prevalence	27	20	22	31
PPV	97	80	96	95
NPV	98	99	95	98

Note: PPV = positive predictive value; NPV = negative predictive value.

**Table 4:** Data for misclassified cases (*n* = 10).

Participant ID Age/Category	Absolute Values; Peak Flow (L/min), TUG (s); Decrement (Log), Anterior Thigh Muscle Thickness (mm); Percentage Thigh Muscle (%); PASE
Y044, 28, young male misclassified as young female	Peak flow = 470; TUG = 6.8; BB Decrement = 1.06; Anterior thigh muscle thickness = 29.9; % thigh muscle = 69
Y051, 25, young female misclassified as older female	Peak flow = 290; TUG = 4.5; BB Decrement = 1.08; Anterior thigh muscle thickness = 19.2; % thigh muscle = 56
Y053, 21, young male misclassified as young female	Peak flow = 450; TUG = 4; BB Decrement = 0.81; Anterior thigh muscle thickness = 25.4; % thigh muscle = 84
OL001, 68, older male misclassified as young female	Peak flow = 510; TUG = 4.9; BB Decrement = 1.25; Anterior thigh muscle thickness = 25.1; % thigh muscle = 68; PASE = 308
OL008, 70, older male misclassified as young female	Peak flow = 300; TUG = 4.9; BB Decrement = 1.22; Anterior thigh muscle thickness = 24.9; % thigh muscle = 81; PASE = 136
OL013, 67, older male misclassified as young male	Peak flow = 810; TUG = 4.4; BB Decrement = 1.47; Anterior thigh muscle thickness = 29.7; % thigh muscle = 85; PASE = 169
OL028, 75, older male misclassified as old female	Peak flow = 370; TUG = 7.1; BB Decrement = 1.13; Anterior thigh muscle thickness = 24.1; % thigh muscle = 52; PASE = 148
OL034, 76, older female misclassified as young female	Peak flow = 400; TUG = 4; BB Decrement = 1.63; Anterior thigh muscle thickness = 28.9; % thigh muscle = 65; PASE = 200
OL040, 73 older male misclassified as young female	Peak flow = 210; TUG = 4.3; BB Decrement = 1.11; Anterior thigh muscle thickness = 32.7; % thigh muscle = 73; PASE = 252
OL076, 73, older female misclassified as old male	Peak flow = 370; TUG = 6.9; BB Decrement = 1.44; Anterior thigh muscle thickness = 22.7; % thigh muscle = 69; PASE = 135

Note: BB = Biceps Brachii; TUG = timed up and go; PASE = physical activity scale for the elderly.

**Table 5:** Correlations between BMI and classification features.

Classification Feature	Correlation
SF-36	−0.22
Physical function	
Grip strength (kg)	−0.15
Quadriceps strength (*N*)	−0.05
Peak flow (L/min)	−0.22
Timed up and go (s)	0.21
Stair climbing time (s)	0.36
Ultrasound Imaging	
Non-contractile tissue thickness (mm)	0.01
Muscle thickness (mm)	0.01
% Non-contractile tissue	0.41
% Muscle	−0.41
Muscle mechanical properties	
**Biceps Brachii**	
Stiffness (N/m)	0.21
Tone (Hz)	−0.07
Decrement (log)	0.47
**Rectus Femoris**	
Stiffness (N/m)	0.01
Tone (Hz)	−0.21
Decrement (log)	0.36

## Data Availability

The data presented in this study are available on request from the corresponding author. The data are not publicly available due to ethics approval being required for secondary analyses of these data.

## Appendix A

### A.1. Co-Morbidities

The majority (*n* = 40, 55%) of older participants in the present study reported the presence of one medical condition (arthritis, high blood pressure, diabetes, irritable bowel syndrome), and 13 (18%) had no history of a medically diagnosed condition (Table A1).

**Table A1:** Percentage of older participants with medical conditions.

Medical Conditions Reported		Older Adults (*n*)	Percent (%)	Valid Percent	Cumulative Percent
Valid	0	13	17.8	18.6	18.6
	1	40	54.8	57.1	75.7
	2	16	21.9	22.9	98.6
	3	1	1.4	1.4	100.0
	Total	70	95.9	100.0	
	Missing	3	4.1		
Total		73	100.0		

### A.2. Information on Use of Prescribed Medication in Older Adults

The majority (*n* = 22, 30%) of older participants in the present study reported using one form of prescribed medication for the conditions mentioned above, and 19 (26%) were not using any form of medication (Table A2).

**Table A2:** Percentage of older participants taking medications.

Number of Medications		Older Adults (*n*)	Percent (%)	Valid Percent	Cumulative Percent
Valid	0	19	26.0	27.1	27.1
	1	22	30.1	31.4	58.6
	2	16	21.9	22.9	81.4
	3	7	9.6	10.0	91.4
	4	3	4.1	4.3	95.7
	5	2	2.7	2.9	98.6
	6	1	1.4	1.4	100.0
	Total	70	95.9	100.0	
	Missing	3	4.1		
Total		73	100.0		
